# Resveratrol Effect on α-Lactalbumin Thermal Stability

**DOI:** 10.3390/biomedicines12102176

**Published:** 2024-09-25

**Authors:** Aurica Precupas, Daniela Gheorghe, Anca Ruxandra Leonties, Vlad Tudor Popa

**Affiliations:** “Ilie Murgulescu” Institute of Physical Chemistry, Romanian Academy, Splaiul Independentei 202, 060021 Bucharest, Romania; chiscan_danny@icf.ro (D.G.); aleonties@icf.ro (A.R.L.)

**Keywords:** lactalbumin, resveratrol, aggregates, unfolding, DSC, CD

## Abstract

The effect of resveratrol (RESV) on α-lactalbumin (α-LA) thermal stability was evaluated using differential scanning calorimetry (DSC), circular dichroism (CD) and dynamic light scattering (DLS) measurements. Complementary information offered by molecular docking served to identify the binding site of the ligand on the native structure of protein and the type of interacting forces. DSC thermograms revealed a double-endotherm pattern with partial overlapping of the two components. The most relevant effect of RESV is manifested in the narrowing of the protein thermal fingerprint: the first process (peak temperature *T*_1_) is shifted to higher temperatures while the second one (peak temperature *T*_2_) to lower values. The CD data indicated partial conformational changes in the protein non-α-helix domain at *T*_1_, resulting in a β-sheet richer intermediate (BSRI) with an unaffected, native-like α-helix backbone. The RESV influence on this process may be defined as slightly demoting, at least within DSC conditions (linear heating rate of 1 K min^−1^). On further heating, unfolding of the α-helix domain takes place at *T*_2_, with RESV acting as a promoter of the process. Long time incubation at 333 K produced the same type of BSRI: no significant effect of RESV on the secondary structure content was detected by CD spectroscopy. Nevertheless, the size distribution of the protein population obtained from DLS measurements revealed the free (non-bound) RESV action manifested in the developing of larger size aggregates.

## 1. Introduction

α-lactalbumin (α-LA), one of the major whey proteins, has received significant interest due to its unique properties and potential therapeutic applications. The structure of α-LA presents two lobes separated by a cleft. One lobe consists of α-helices, whereas the other comprises β-sheets and unordered structures [1]. An essential characteristic of α-LA is its ability to bind metal cations [2]. Calcium binding enables protein stability and is an important factor for proper folding [3]. The primary calcium binding site in α-LA connects the two lobes [4]. Recent studies revealed potential therapeutic effects of α-LA in various health conditions: metabolic disorders, neurodegenerative diseases, cancer [5,6,7]. Bactericidal activity of α-LA has also been reported [8]. The protein interacts with other bioactive compounds with various functional characteristics involved in food science [9,10]. To acquire a better understanding of bioactive compound transport and metabolism, it is necessary to explore the interactions between these ligands and carrier proteins. The interaction of milk proteins with biologically active molecules is an important aspect in the development of dairy products and healthy diets. The interaction of α-LA with retinol and palmitic acid [9] shows the potential of using α-LA for the encapsulation of hydrophobic compounds. α-LA binds and transports drugs such as vitamin D, genistein, kaempferol, and EGCG with high affinity [11,12,13].

Protein aggregation involves the occurrence of conformation changes due to misfolding, which further results in aggregates or fibril formation [14]. A significant number of human diseases are linked to protein aggregation, generating interest not only in medical sciences but also in biochemistry, biophysics, and biotechnology. Although α-LA is not directly involved in any amyloidosis disease, it is a model protein for misfolding and aggregation studies as it can form different folding intermediates under varying conditions. The literature studies report the folding and unfolding of α-LA at different pH values. Apo α-LA (calcium-depleted form) was reported to undergo conformational changes caused by calcium removal that results in aggregate formation near the protein’s characteristic pI (4.0–5.0) [15,16,17]. Protein aggregation is also an important characteristic associated with food processing, closely related to dairy product stability and quality. The aggregation behavior of whey proteins might modify their viscosity, foaming, emulsification, and antioxidant and antibacterial properties. The understanding of the heat-induced unfolding, aggregation and gelation mechanisms under different environmental conditions is important for the development of various functional foods. Various types of molecules, of natural and synthetic origin, were tested (in vitro and in vivo) to estimate their anti-amyloid action. The most promising results were obtained with several natural molecules, particularly polyphenols with a proven efficacy against protein aggregation. Among natural polyphenols, resveratrol was chosen for two reasons: (i) its influence on protein thermal stability is less studied; and (ii) there are several documented applications of RESV in medical and food science, particularly in connection/interaction with whey proteins. The main problem in RESV utilization consists of its low aqueous solubility that limits bioavailability. Binding to carrier proteins is a feasible solution to this problem and allows for higher serum concentration of resveratrol. Whey proteins like α-lactalbumin are good carrier candidates due to their binding affinity for RESV. Moreover, they exhibit good nutritional features at a low price.

Polyphenol–protein interaction has received much attention in recent years. Whey proteins are involved in noncovalent interactions with polyphenols and serve as delivery vehicles. Different studies reported the interaction of polyphenols with casein [18], bovine serum albumin (BSA) [19,20,21], lysozyme [22], β-lactoglobulin [23], and α-lactalbumin [24,25]. Our previous investigations revealed that polyphenolic compounds present various effects on protein thermal stability in a concentration-dependent manner [26,27,28]. Polyphenols present a research interest due to their health beneficial effects, especially in the treatment and prevention of cancer and cardiovascular diseases [29]. One important aspect of the interaction between natural polyphenolic compounds and milk proteins consists of its reciprocal effects: protein–phenol interactions might modify bioavailability of both proteins and phenols [30]. Resveratrol (3,5,4′-trihydroxy-trans-stilbene), RESV, is a naturally occurring phenolic compound found in plants with numerous health benefits: antioxidant, cardioprotective, anticancer and anti-inflammatory effects [31]. Resveratrol binds to β-lactoglobulin without changing the protein secondary structure. A non-specific binding of resveratrol on the surface of the protein and self-association of resveratrol at high concentrations were proposed [32]. The interaction of bovine α-lactalbumin with curcumin and trans-resveratrol [33] was investigated by fluorescence-quenching measurements and docking studies. Trans-resveratrol binds in the vicinity of Trp-60 and Trp-104 of α-LA, with the binding stoichiometry of 1:1, and hydrogen bonds contribute to the stability of the complex. Therefore, the present study focuses on the interactions between α-LA and RESV and investigates the effect of resveratrol on the thermal stability of α-LA using DSC, CD spectroscopy, and dynamic light scattering. Moreover, molecular docking provided the probable binding site of the ligand to the protein. This research presents a novel approach for evaluating α-LA thermal stability in time, at different temperatures and ligand concentrations that may be used to develop novel aggregation inhibitors and anti-amyloid therapeutics.

## 2. Materials and Methods

### 2.1. Materials

Bovine lactalbumin, α-LA, (L7252, lyophilized powder, CAS: 9013-90-5, Sigma-Aldrich, Saint Louis, MO, USA) and trans-resveratrol, RESV, (Evolva SA, Reinach, Switzerland, purity > 98%) were used with no further purification. Solutions of the protein and RESV were prepared in 30 mM of phosphate buffer (KH_2_PO_4_, K_2_HPO_4_) at pH 7.2. For DSC, CD, and DLS measurements, the samples of α-LA and RESV are as follows: α-LA at molar ratios 1:1 and 3:1 (Table 1) were allowed to equilibrate for 24 h at 277 K and brought to room temperature before measurements. Furthermore, for CD measurements, the samples of protein (1.43 × 10^−4^ M) and RESV: α-LA at molar ratios 1:1 and 3:1 were kept at 333 K in an incubator without stirring for different time intervals (24 h and 48 h). Also, the CD measurements were performed for the same systems after 24 h incubation at 277 K and their thermal stability was evaluated at 222 nm with increasing temperature.

### 2.2. Methods

#### 2.2.1. Differential Scanning Calorimetry (DSC)

The thermal stability of α-LA and the influence of RESV were evaluated using a Nano DSC differential scanning calorimeter from TA Instruments (NanoDSC III, TA Instruments, Lindon, UT, USA) in a temperature range of 293–373 K, at constant pressure of 2 atm, using a scan rate of 1 K min^−1^. The reference cell was filled with 30 mM of buffer phosphate, pH 7.2. The solutions were degassed for 10 min under vacuum in a degassing station (TA Instruments) at room temperature and then immediately injected into the calorimeter cells. The protein concentration was constant (1.43 × 10^−4^ M), while the mixtures of ligand–protein were prepared at two different concentrations of RESV: 1.43 × 10^−4^ M and 4.28 × 10^−4^ M. For RESV: α-LA systems, the excess heat capacity scans were determined after subtracting the DSC signal of RESV at the corresponding concentration, and the difference in heat capacity between the initial and the final state was corrected using a sigmoidal 1st order baseline via TA NanoAnalyze v. 2.4.1 software, as previously described [34,35]. The denaturation enthalpy change, Δ*H_cal_*, temperature, *T*, and entropy change, ∆*S*, were estimated using the instrument software. Assuming two transitions in the denaturation pathway, the DSC thermograms were decomposed using PeakFit v.4.12 software with Haarhoff–Van der Linde built-in function [36].

#### 2.2.2. Circular Dichroism (CD)

Incubation of α-LA at 333 K results in the (partial) unfolding of the globular structure of the protein molecule that can form aggregates with other unfolded monomers [37]. CD measurements were performed to evaluate the changes in the secondary structures of α-LA and RESV:α-LA systems after incubation at 277 K (for 24 h) and 333 K (for 24 h and 48 h). A JASCO J-815 spectropolarimeter (Jasco, Japan), equipped with a Peltier-type temperature controller was used to obtain the far-UV CD spectra of protein using a 1 cm quartz cuvette, at 298 K, with three accumulations for each measurement. The protein concentration (0.4 μM) was maintained constant. The time constant, scan speed, resolution, and sensitivity were set at 1 s, 100 nm min^−1^, 1.0 nm, and 100 mdeg, respectively. The CD spectra of protein samples were carried out using a 30 mM phosphate buffer, pH 7.2 as blank. The CD spectra of RESV-protein systems were baseline-corrected by subtracting blank spectra of the corresponding RESV solutions, at the same concentration. The results are expressed as ellipticity (mdeg), and the secondary structure content was obtained from the Dichroweb website [38] using the K2D analysis algorithm [39]. Normalized root-mean-square deviations (NRMSD) lower than 0.2 were obtained for all fits of CD spectra.

The effect of RESV on the α-helix thermal stability of protein was evaluated by monitoring the changes in the CD signal at 222 nm in the temperature range 293–348 K using a heating rate of 1 K min^−1^. The protein and the ligand were mixed at a 1:1 and 3:1 molar ratio, incubated for 24 h at 277 K and equilibrated at room temperature before heating.

#### 2.2.3. Dynamic Light Scattering (DLS)

The average size distribution of α-La in the absence and presence of RESV, for the samples incubated 24 h at 277 K, was determined based on DLS measurements performed on a Zetasizer Nano ZS (Malvern Instruments, Worcestershire, UK). A 0.22 μm pore-sized microfilter was used to filter all of the solutions. Samples of α-LA (1.43 × 10^−4^ M) in a 30 mM phosphate buffer, pH 7.2 at different concentrations of RESV, were loaded in standard disposable cuvettes, and the average hydrodynamic diameter (*D_h_*) and the polydispersity index (PDI), the indicator of the homogeneity of the solution, were determined. Experiments were conducted at 298 K and the results are the average of five measurements. The results were reported in terms of the number average of particle size.

#### 2.2.4. Molecular Docking

The binding of RESV with α-LA was studied using molecular docking. The holo-form (calcium-containing) protein structure (PDB ID: 1F6S) [40] was obtained from the RCSB Protein Data Bank [41]. The geometry of trans-resveratrol was optimized by the DFT/B3LYP/6-311G++ level of theory using the Gaussian03 software [42], as previously described [43]. Molecular docking calculations were performed using the Autodock Vina 1.1.2 software [44]. Autodock tools [45] were used for protein and ligand file preparation to add all hydrogen atoms, to assign the Gasteiger charges, and to detect and assign the rotatable bonds of the ligand. The Lamarckian genetic algorithm with a population size of 150 and maximum number of 2,500,000 energy evolutions were selected to determine the optimum binding site of the RESV to the protein, set as rigid. The ligand was allowed for flexible rotation to obtain a possible conformation that binds to the protein. The grid size was set to cover the whole protein at 54, 40, 40 points along x, y, and z axes, centered on x, y, and z coordinates of 43 Å × 90 Å × 10 Å, using a grid point spacing of 1 Å. The lowest energy conformation of the ligand–protein complex was analyzed. The type of interaction was evaluated using the BIOVIA Discovery studio 2019 [46].

## 3. Results and Discussion

### 3.1. Thermal Stability of Protein in the Absence and Presence of RESV

**Figure 1:**
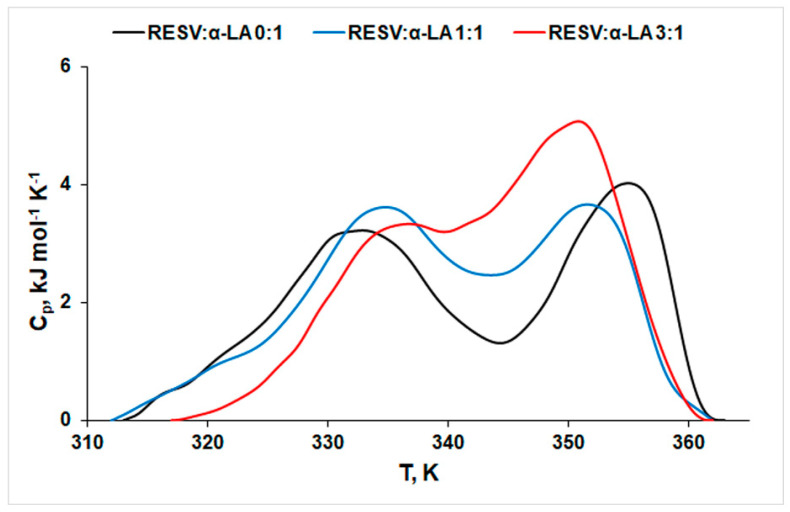
DSC scans of α-LA denaturation in the absence and presence of RESV at different protein:ligand molar ratios in 30 mM phosphate buffer, pH 7.2.

The thermal stability of α-LA was evaluated in the absence and presence of RESV using DSC measurements. The thermograms pertaining to different concentrations of resveratrol and revealing its influence on α-LA thermal stability are presented in Figure 1. The DSC signals were decomposed in PeakFit^®^ (Figure S1 in Supplementary Materials) and the thermodynamic parameters of denaturation are summarized in Table 2.

The thermograms of protein thermal denaturation display two endothermic, partially overlapping peaks, both in the absence and presence of RESV. Two domains with a significantly different conformational stability were reported for α-LA unfolding [47]: while lobe 1 unfolds at lower temperatures, lobe 2 contains the thermal stabilizer Ca^2+^ that shifts its unfolding to a higher temperature. The protein unfolding occurs due to a disruption of van der Waals forces and hydrogen bonds that stabilize the protein structures, followed by hydration of the exposed residues [48]. A simple, naked eye inspection of Figure 1 reveals a two-fold action of RESV on protein thermal stability:

(1) On one side, the peak corresponding to the first (lower temperature, *T*_1_) process shifts to higher values, indicating a stabilizing effect of RESV. The same effect on protein structure was reported for doxorubicin [49].
(2) On the other side, the temperature values corresponding to the second peak (*T*_2_) decrease with increasing concentration of RESV, pointing to a destabilization effect of both bound and free forms of the ligand on the protein structure. One may notice that RESV action on this second process is more pronounced than its action on the first process (|Δ*T*_1_| = 2.6 K, |Δ*T*_2_| = 4.2 K). Higher values of ∆*H_cal_* obtained in the presence of RESV indicate a lower exposure of the hydrophobic regions of the native protein to the solvent molecules, during the unfolding process in the presence of RESV [19]. The positive values of Δ*S* sign for rearrangements within the protein molecule in the unfolding process.

### 3.2. Changes in the Secondary Structure of Protein

**Figure 2:**
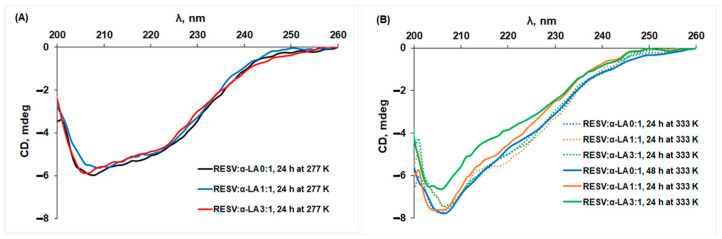
The CD spectra of α-LA after incubation at (**A**) 24 h at 277 K and (**B**) for different time intervals at 333 K, in the absence and presence of different concentration of RESV.

The changes in the secondary structure of protein as result of various incubation time intervals at different temperatures, in the absence and presence of RESV were evaluated by CD measurements (Figure 2).

**Figure 3:**
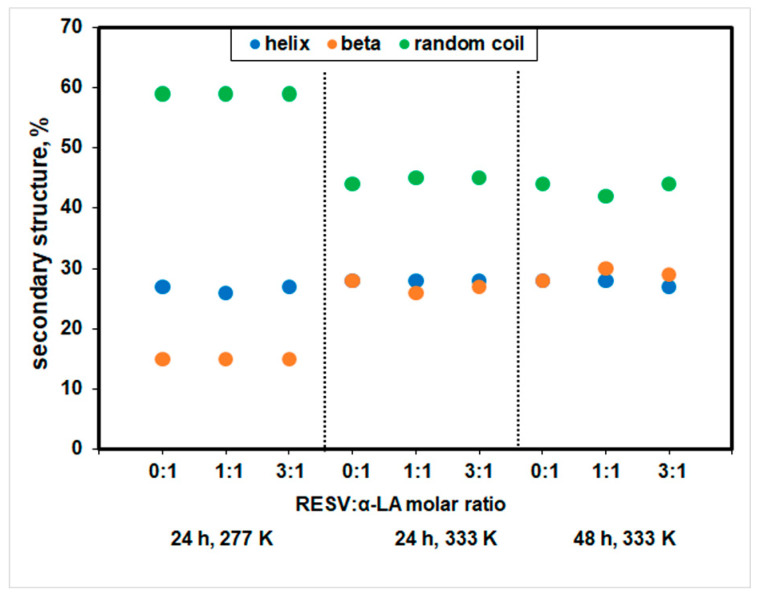
The secondary structure content of α-LA at different incubation time intervals and temperatures, in the absence and presence of RESV.

The CD spectra of α-LA exhibit two negative signals at 208 and 222 nm, characteristic of the typical α-helix structure of proteins [50] for the samples incubated at 277 K for 24 h (Figure 2A). The shape of the CD spectra for α-LA changes with temperature, and an increase in the negative value at 208 nm was obtained at 333 K (Figure 2B), while the secondary structure content remains constant, as the experimental result analysis by Dichroweb indicated (Figure 3).

A Dichroweb CD data analysis displayed a secondary structure content of 27% α-helix, 15% β and 59% random coil structures for α-LA after a 24 h incubation at 277 K, in good agreement with the values reported in the literature [51]. For α-LA, incubation at 333 K for 24 h and 48 h led to a significant increase in β structure and a pronounced loss of random coil, and a β-sheet richer intermediate (BSRI) with unaffected, native-like α-helix backbone was obtained. No significant change in the protein secondary structure content for all incubation time intervals was achieved in the presence of the ligand. A similar effect was reported at 298 K for capsaicin binding to holo-α-LA [52].

The most important feature displayed in Figure 3 consists of the practically unchanged content of α-helix, whatever the incubation time and temperature, in both the presence and absence of resveratrol. Based on CD data, one may positively ascribe the first process displayed in DSC thermograms to conformational rearrangements involving non-α-helix domains of the protein: β content increases at the expense of random coil, while the α-helix backbone remains practically untouched, resulting in a β-sheet-rich intermediate (BRSI) with unchanged, native-like α-helix content. The second, higher temperature endotherm process displayed in the DSC thermograms may thus confidently be ascribed to the unfolding of the α-helix structure. A partially unfolded state of Ca^2+^-loaded α-LA at 343 K was reported in the literature [53].

**Figure 4:**
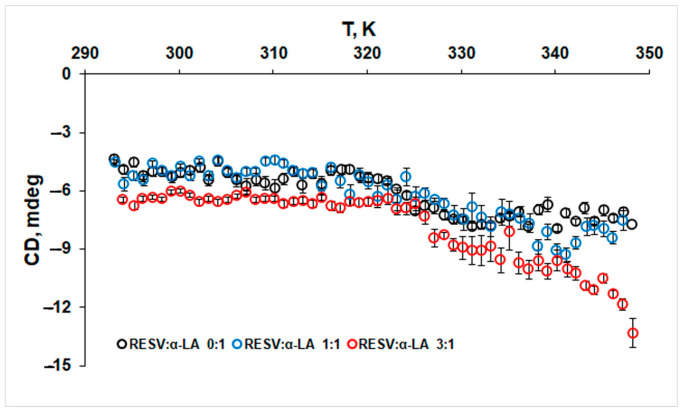
CD thermal denaturation curves of α-LA, pH 7.2, in the absence and presence of RESV. Samples were heated at the scan rate of 1 K min^−1^, and CD values (±standard deviation) were monitored at 222 nm.

Additionally, the thermal stability of the α-helix structure was evaluated by monitoring the CD signal at 222 nm (Figure 4).

The negative value of ellipticity at 222 nm corresponding to the α-helix structure of protein slightly increases after 330 K, both in the absence and presence of RESV. This result is in good agreement with the DSC measurements that indicated the unfolding of α-helix structure at higher temperatures.

### 3.3. Dynamic Light Scattering

**Figure 5:**
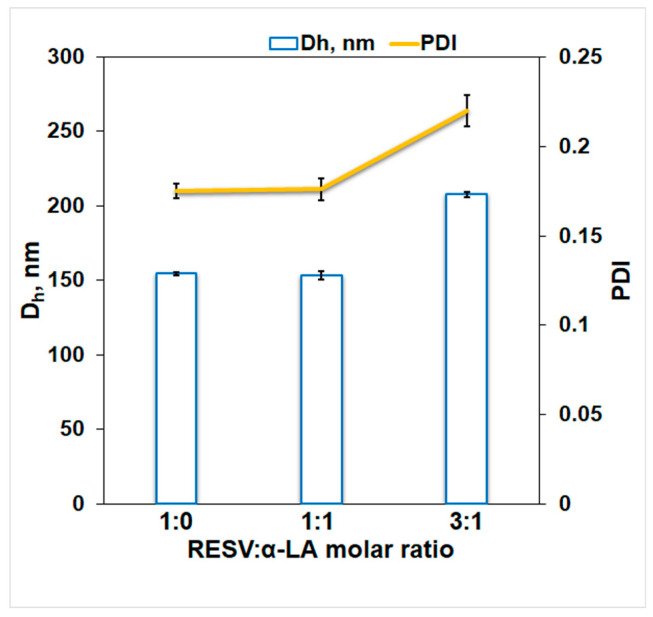
Hydrodynamic diameter and polydispersity index for different RESV: α-LA molar ratios after 24 h’ incubation at 277 K.

The size distribution by number for RESV: α-LA systems after a 24 h incubation at 277 K (Figure S2 in Supplementary Materials) presents a single peak corresponding to aggregates with a mean size of ~155 nm for RESV: α-LA 0:1 and 1:1 molar ratios (Figure 5).

The size of α-LA aggregates is in good agreement with the value obtained for bovine α-lactalbumin studied in interaction with oleuropein [54]. RESV induces protein aggregation in a concentration-dependent manner. For a 1:1 RESV:α-LA molar ratio, no significant change in the protein aggregate size was observed. Meanwhile, a higher concentration of RESV promotes protein aggregation, the *D_h_* value increases to 208 nm for a RESV: α-LA 3:1 molar ratio. The higher value of PDI obtained for an increasing concentration of the ligand suggests the presence of larger size aggregates. The results point to a dual action of RESV on protein aggregation: the bound ligand stabilizes the size of the protein aggregates, while the free RESV promotes aggregation. The increase in PDI and *D_h_* values evidences the free ligand effect on protein aggregation.

### 3.4. Molecular Docking

**Figure 6:**
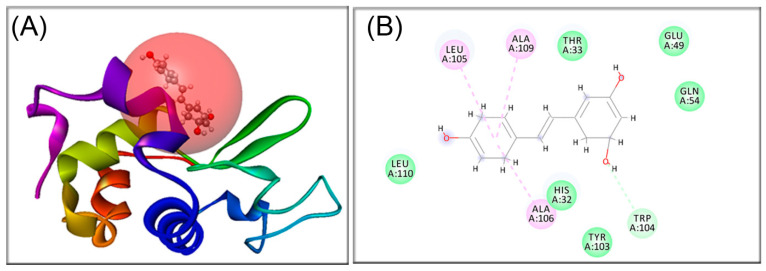
Molecular docking of RESV and α-LA; (**A**) RESV is presented with a stick-and-balls model and the protein as solid ribbon. (**B**) The 2D representation of RESV–α-LA complex; the close amino acid residues are presented in green, dashed lines represent intermolecular interactions of different origin (alkyl–alkyl—pink lines; hydrogen bond—green line; van der Waals interactions—light green lines).

The binding site of RESV within the native structure of the protein and the interacting forces were predicted using molecular docking (Figure 6A).

A molecular docking analysis shows that RESV binds with α-LA with a significant binding affinity of −6.1 kcal/mol, similar to the values obtained for the interaction with another phenolic compounds, oleuropein [54] and ellagic acid [55]. Figure 6B depicts the two-dimensional diagram of α-LA residues interacting with RESV by hydrogen bond (TRP104, 3.49 Å) and hydrophobic interactions with LEU105 (5.43 Å), ALA106 (3.88 Å), and ALA109 (4.98 Å). The bound ligand’s pose within the non-α-helix domain of the protein accounts for its action on the lower temperature (*T*_1_) conformational changes. Once the stable BSRI is formed, one may suspect that the promoting α-helix denaturation effect of RESV is exerted in its unbound state.

The results of this investigation of the resveratrol effect on the thermal stability of α-LA using DSC, CD spectroscopy, dynamic light scattering, and molecular docking revealed unexplored features of protein—RESV interaction, providing a deeper insight into the complex action of natural, biologically active polyphenolic compounds. The embodied information may thus be utilized prior to clinical studies, to develop novel aggregation inhibitors and anti-amyloid therapeutics. Combined with their properties of bioactive carriers, milk proteins display intrinsic nutritional functions. Therefore, the results of the present study (and subsequent ones) are useful for food science and technology.

## 4. Conclusions

The effect of resveratrol binding on α-lactalbumin thermal stability was evaluated by DSC, CD, DLS, and molecular docking. DSC thermograms evidenced bi-modal endotherm behavior with denaturation temperatures *T*_1_ ~ 333–336 K and *T*_2_ ~ 351–355 K, with partial overlapping of the two peaks. This thermal fingerprint suggests the formation of a (more or less) stable intermediate. The chosen (333 K) incubation temperature was meant to evidence the presence of this intermediate via CD.

Corroboration of DSC and CD results confirmed the existence of this intermediate and clarified the dual effect of the ligand on the protein thermal stability: (1) RESV demotes, i.e., delays (on the T scale) conformational alteration of the non-alpha-helix domain of the protein, with the formation of a stable β-sheet-rich intermediate (BSRI); (2) it acts as a promoter for the denaturation of the α-helix domain that takes place at *T*_2_. The CD data indicated the negligible influence of RESV content on the secondary structure composition of samples incubated at 333 K for 24 and 48 h.

The DLS measurements revealed the protein aggregation taking place after 24 h incubation at 277 K. In equimolar concentration, RESV does not influence the aggregation process. At a 3:1 molar ratio, a manifest increase in *D_h_* and PDI takes place, indicating the aggregation promoting the action of free (not protein-bound) ligand.

The binding site of RESV on native protein and the interacting forces were estimated by molecular docking that further complemented the in vitro observations and provided structural insight into the interaction RESV with α-LA.

Future research must expand the range of proteins and polyphenols, with emphasis on various protein–polyphenol assemblies for the potential use in biomedical fields and/or in the food industry.

## Figures and Tables

**Table 1:** The studied ligand–protein systems.

Ligand–Protein System	RESV Concentration, M	α-LA Concentration, M
RESV:α-LA 0:1	0	1.43 × 10^−4^
RESV:α-LA 1:1	1.43 × 10^−4^	1.43 × 10^−4^
RESV:α-LA 3:1	4.28 × 10^−4^	1.43 × 10^−4^

**Table 2:** Thermal denaturation parameters (±S.E.) obtained from PeakFit decomposition of the α-LA thermal signal in the absence and presence of different concentrations of RESV. S.E. represents standard error of the fitting parameters given by PeakFit. Last two rows contain the overall values of the thermodynamic quantities offered by NanoAnalyze (instrument) v4.0.2 software.

Thermodynamic Parameters	RESV:α-LA 0:1	RESV:α-LA 1:1	RESV:α-LA 3:1
*T*_1_, K	333.14 ± 0.07	334.05 ± 0.09	335.74 ± 1.32
*T*_2_, K	355.20 ± 0.05	352.04 ± 0.16	350.99 ± 0.42
∆*H*_1_, kJ mol^−1^	54.69 ± 0.07	43.54 ± 0.22	58.18 ± 3.17
∆*H*_2_, kJ mol^−1^	45.37 ± 0.06	57.86 ± 0.22	47.75 ± 2.55
∆*H_cal_*, kJ mol^−1^	100.33	101.40	105.93
∆*S*, kJ mol^−1^ K^−1^	0.283	0.289	0.312

## Data Availability

The data are contained within this article and the Supplementary Materials.

## Supplementary Materials

The following supporting information can be downloaded at: https://www.mdpi.com/article/10.3390/biomedicines12102176/s1, Figure S1: PeakFit decomposition of DSC thermograms for (A) α-LA, (B) RESV:α-LA 1:1 molar ratio and (C) RESV:α-LA 3:1 molar ratio; Figure S2: size distribution by number for RESV: α-LA 0:1 (red line), RESV: α-LA 1:1 (green line), and RESV: α-LA 3:1 (blue line) after a 24 h incubation at 277 K.
