# Supplementary material for: Resveratrol Effect on α-Lactalbumin Thermal Stability
Source: Biomedicines. 2024 Sep 25;12(10):2176. doi: 10.3390/biomedicines12102176 (PMC11504486; doi:10.3390/biomedicines12102176)
Supplement: Supplementary file 1 [file biomedicines-12-02176-s001.zip › biomedicines-3208651-supplementary.pdf]

Supplementary Material

Resveratrol Effect on α-Lactalbumin Thermal Stability

Aurica Precupas *, Daniela Gheorghe, Anca Ruxandra Leonties and Vlad Tudor Popa *

"Ilie Murgulescu" Institute of Physical Chemistry, Romanian Academy, Splaiul Independentei 202, 060021 Bucharest, Romania
* Correspondence: aprecupas@icf.ro (A.P.); vtpopa@icf.ro (V.T.P.)

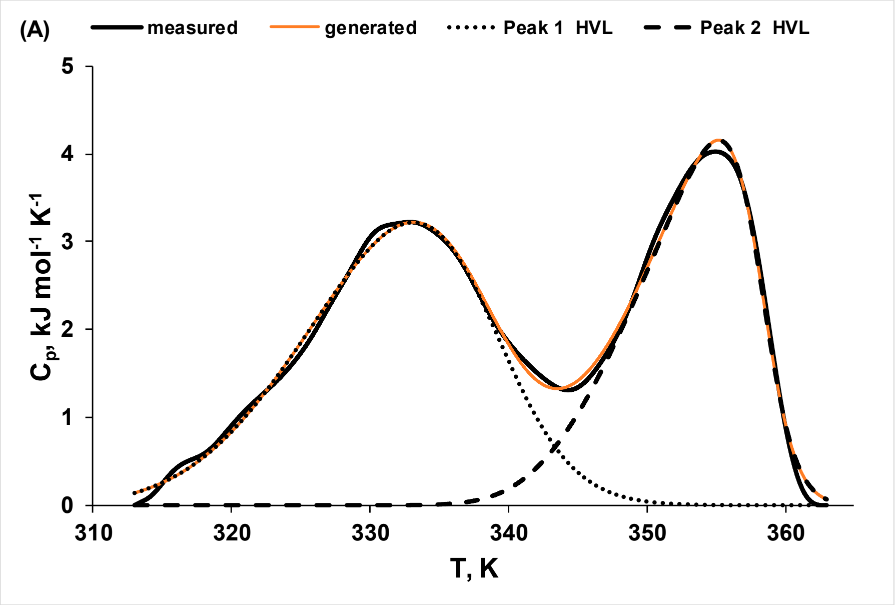

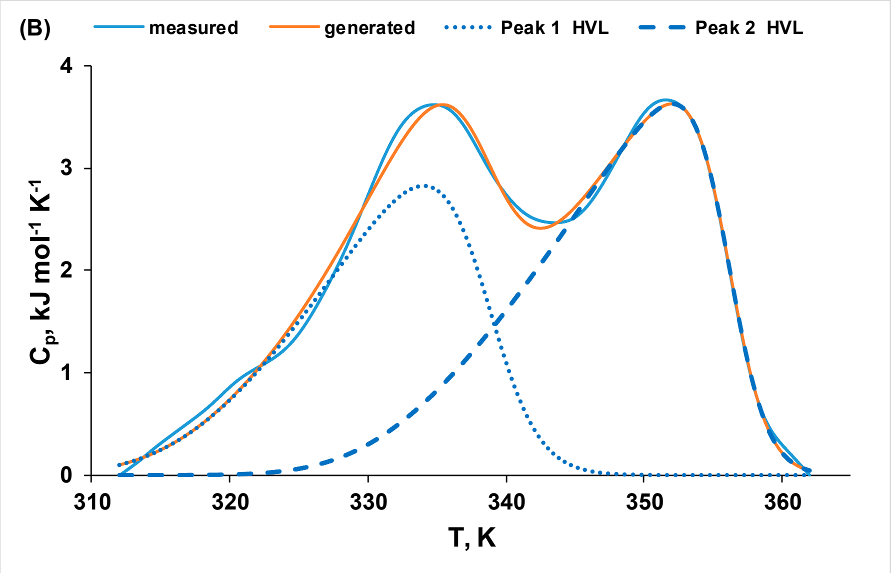

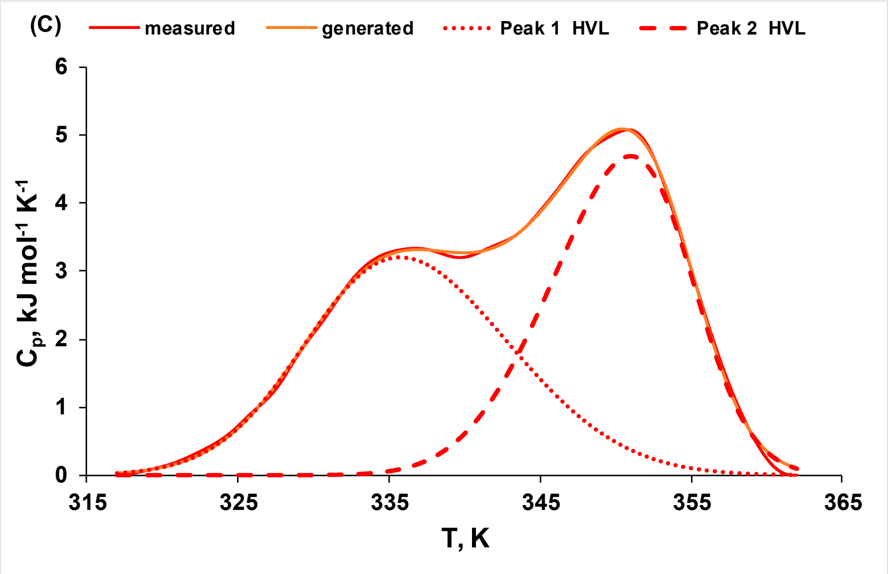

Figure S1. PeakFit decomposition of DSC thermograms for (A) α-LA, (B) RESV:α-LA 1:1 molar ratio and (C) RESV:α-LA 3:1 molar ratio.

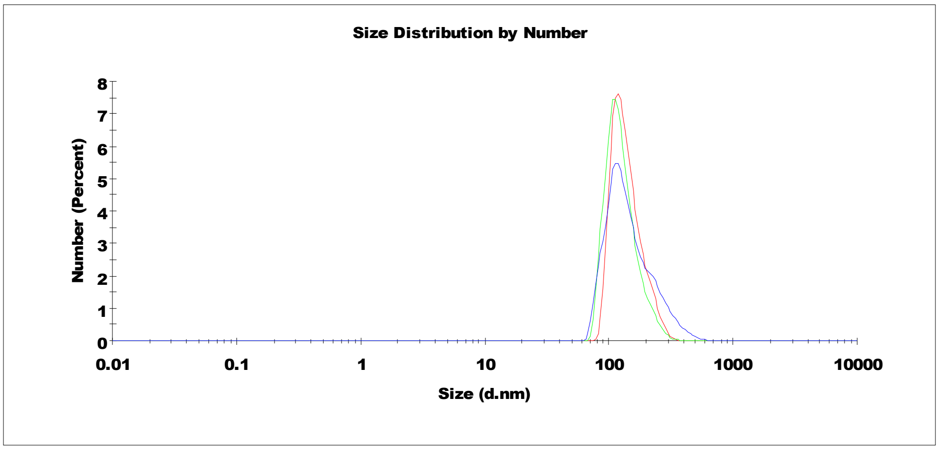

Figure S2. Size distribution by number for RESV: α-LA 0:1 molar ratio (red line), RESV: α-LA 1:1 molar ratio (green line) and RESV: α-LA 3:1 molar ratio (blue line) after 24 h incubation at 277 K.
